# An open source extrusion bioprinter based on the E3D motion system and tool changer to enable FRESH and multimaterial bioprinting

**DOI:** 10.1038/s41598-021-00931-1

**Published:** 2021-11-03

**Authors:** Adam Engberg, Christina Stelzl, Olle Eriksson, Paul O’Callaghan, Johan Kreuger

**Affiliations:** Department of Medical Cell Biology, Uppsala University, Uppsala, Sweden

**Keywords:** Tissue engineering, Assay systems

## Abstract

Bioprinting is increasingly used to create complex tissue constructs for an array of research applications, and there are also increasing efforts to print tissues for transplantation. Bioprinting may also prove valuable in the context of drug screening for personalized medicine for treatment of diseases such as cancer. However, the rapidly expanding bioprinting research field is currently limited by access to bioprinters. To increase the availability of bioprinting technologies we present here an open source extrusion bioprinter based on the E3D motion system and tool changer to enable high-resolution multimaterial bioprinting. As proof of concept, the bioprinter is used to create collagen constructs using freeform reversible embedding of suspended hydrogels (FRESH) methodology, as well as multimaterial constructs composed of distinct sections of laminin and collagen. Data is presented demonstrating that the bioprinted constructs support growth of cells either seeded onto printed constructs or included in the bioink prior to bioprinting. This open source bioprinter is easily adapted for different bioprinting applications, and additional tools can be incorporated to increase the capabilities of the system.

## Introduction

Additive manufacturing is an umbrella term for a variety of manufacturing techniques usually based on building three-dimensional (3D) objects in a layer-by-layer fashion. Additive technologies are gaining widespread popularity in a variety of research fields, as well as in industry, as 3D printing enables rapid prototyping and generation of objects with complex geometries from a very wide range and combination of materials. Bioprinting describes the application of additive manufacturing to reproducibly create tissue-like 3D constructs composed of biomolecules and/or biomaterials and cells. As a tissue engineering technology, bioprinting permits cells to be incorporated into printed constructs post-production or during the printing process, and the ultimate goal is often to recapitulate the architecture and function of living tissues and organs^1,2^. Such constructs present opportunities to study cell biology and pathogenic processes in complex, well-defined environments. Bioprinted constructs also hold potential to become widely used model systems and assays for drug screening^3–5^, and their viability as transplantable materials for patient care is an area of intense research interest^6–9^.

Developments in the field of bioprinting encompasses many established fields of research such as material science, cell and tissue engineering, regenerative medicine, as well as robotics, automation and biotechnology. This requirement for interdisciplinary collaborations presents a number of challenges to progress in the bioprinting field; however, by increasing the availability of user-friendly solutions, more researchers are likely to adopt the technology and in doing so advance the field as a whole. As with standard additive manufacturing, many different technologies are capable of constructing bioprinted 3D-objects in a layer-by-layer fashion. The most commonly used techniques for the printing of cell-laden bioinks are extrusion-based bioprinting^10^, laser-assisted bioprinting, stereolithography, and inkjet-based bioprinting^11^. To date extrusion-based bioprinting is one of the most widespread techniques, which has borrowed innovations from the analogous fused filament fabrication (FFF) technology that is extensively used in plastic additive manufacturing. Compared to other bioprinting technologies, extrusion based bioprinting has several advantages such as low cost, a relatively high printing speed, and compatibility with bioinks in a wide range of viscosities. However, extrusion-based bioprinting does suffer from a relatively low spatial resolution, and cell viability can be compromised due to the mechanical stress generated during extrusion of cells through the deposition needle or nozzle^12^.

A challenge that confronts many additive manufacturing techniques, especially those using soft materials such as hydrogels, is the low structural stability of the construct during the printing process, which can negatively impact the geometry and architecture of the intended construct design. Strategies that have been employed to circumvent this limitation include the use of sacrificial support materials. This allows for a construct to be printed in one or more bioinks of choice, while it is simultaneously embedded in a structurally supportive scaffold material printed in parallel. Once the construct is complete and stabilized through post-printing reactions or treatments, the sacrificial material can be removed to release the construct. This technique can also be used to introduce design features such as channels that facilitate the perfusion of printed constructs^13,14^. However, this strategy employing sacrificial materials typically demands a bioprinting platform with the capacity to perform multimaterial printing. Freeform reversible embedding of suspended hydrogels (FRESH) is an innovative solution that provides a 3D structurally supportive print environment, without the need for multimaterial printer functionality^15,16^. During FRESH bioprinting collagen-based bioinks (or other bioinks of interest) are extruded directly into a support bath consisting of gelatin microparticles, and this permits complex 3D constructs to be printed in an essentially suspended state, without the need for printed support structures. The gelatin bath can subsequently be melted away by increasing the temperature, allowing the construct to be retrieved. The FRESH bioprinting strategy has been widely adopted throughout the bioprinting research community^17^; therefore, designing bioprinting platforms that are compatible with FRESH bioprinting will likely be of great general interest.

Efforts to bring bioprinters to a wider audience have included the repurposing of affordable FFF-printers for bioprinting applications^18,19^, and while they may be limited to the extrusion of a single type of bioink at a time, previous studies demonstrate the benefits of leveraging existing additive manufacturing technologies for bioprinting. In the present study we present an open source extrusion-based bioprinter built around the E3D motion system and tool changer, with the potential to produce constructs using up to four syringe pump tools and bioinks. The bioprinter features a transparent polycarbonate cabinet with an integrated HEPA filter and air intake fan to enable contamination-free bioprinting, and automatic tool offset calibration and bed leveling, making it easy to use multiple syringe pump tools to combine different bioinks in order to create complex bioprinted 3D constructs. Due to the open source nature of the components used, the system is highly adaptable to allow for incorporation of new tools and functionalities. FRESH bioprinting as well as multimaterial printing with collagen and laminin was performed to demonstrate the capacity of the system.

## Results and discussion

### Bioprinter assembly

**Figure 1:**
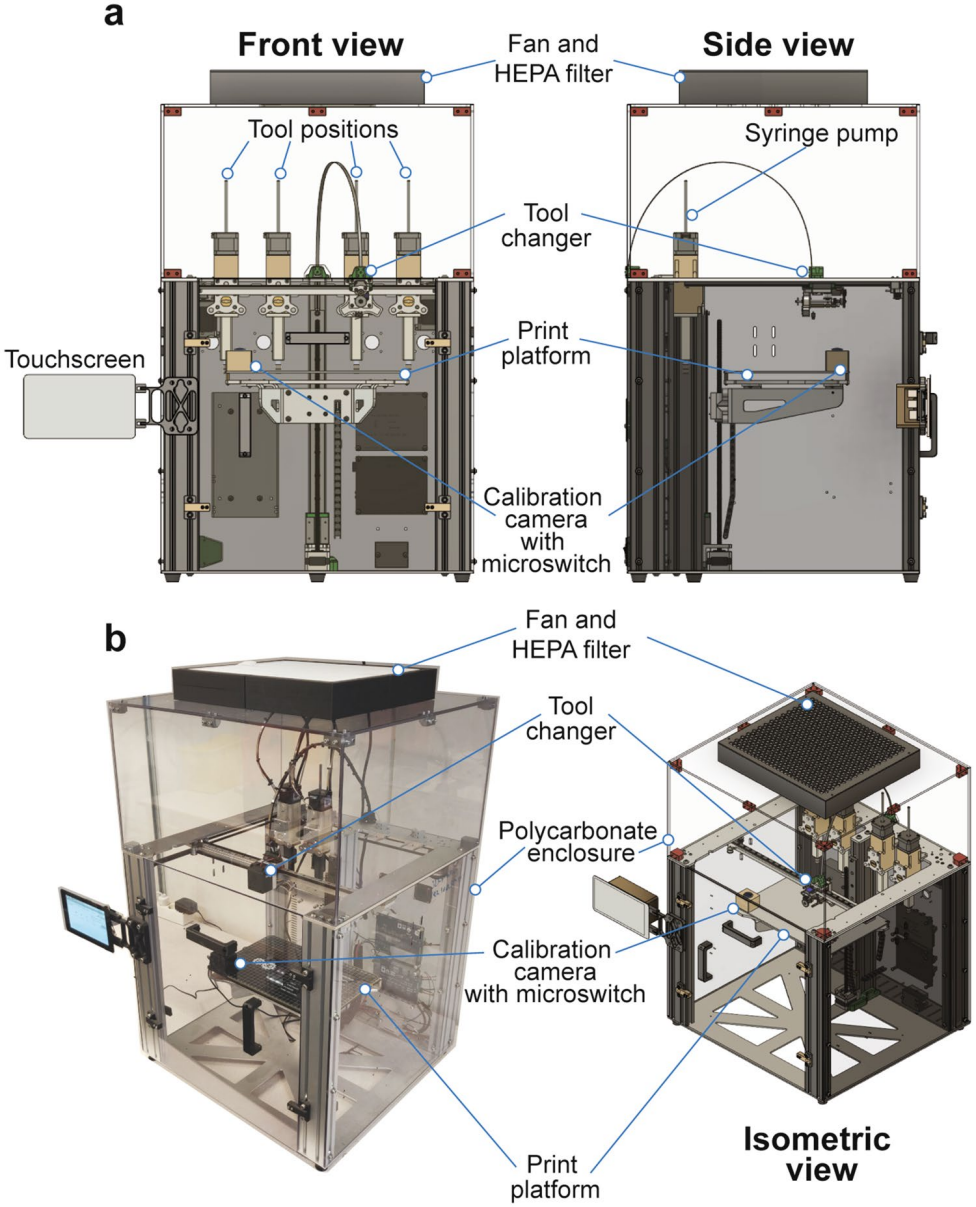
Overview of the open source bioprinter based on the E3D motion system and tool changer. (**a**) Front view and side view CAD illustrations of the open source bioprinter. A transparent polycarbonate box with an integrated HEPA filter with an air intake fan encloses the tool changer to reduce the risk for contamination during the bioprinting process. The tool changer, which is translated along the x- and y-axes, and can retrieve tools from four different tool positions (four syringe pump extrusion tools are shown). The print platform is translated along the z-axis and is equipped with a calibration camera with a microswitch that is used to correct for changes in extrusion position following a change of syringe pump tool. A touch screen attached to the exterior of the cabinet facilitates control of tool selection and calibration functions. (**b**) Isometric views of the open source bioprinter. The left panel presents a photograph of the assembled bioprinter and the right panel presents a CAD illustration.

The open source extrusion bioprinter was built around the E3D motion system and tool changer, and fitted with custom-built syringe pump tools (Fig. 1a). The E3D motion system is easily configurable and delivers robust and precise motion control^20^. E3D provides free access to the systems build files, which facilitated the design process when adapting the existing system for the bioprinting purpose described here^21^. A polycarbonate box compatible with ethanol-based surface sterilization and fitted with an integrated HEPA filter and air intake fan was constructed to enclose the E3D system to reduce the risk of contamination of bioprinted constructs. The polycarbonate enclosure could easily be adapted to allow for temperature and humidity control should this be required for future bioprinting applications (Fig. 1b). The tool changer retrieves tools (in this case syringe pumps) from four positions at the back of the motion system frame, and these tools together enable multimaterial printing. The motion system controls the tools x and y positions, essentially with micrometer precision^20^. The print platform is operated separately and translated along the z-axis. A web camera for x- and y-axis calibration and microswitch for z-axis calibration was mounted on the print platform to enable accurate spatial alignment of tools before multimaterial printing (Fig. 1a,b), and a calibration protocol was operated via a side-mounted touchscreen display.

### Syringe pump extrusion tool design and assembly

**Figure 2:**
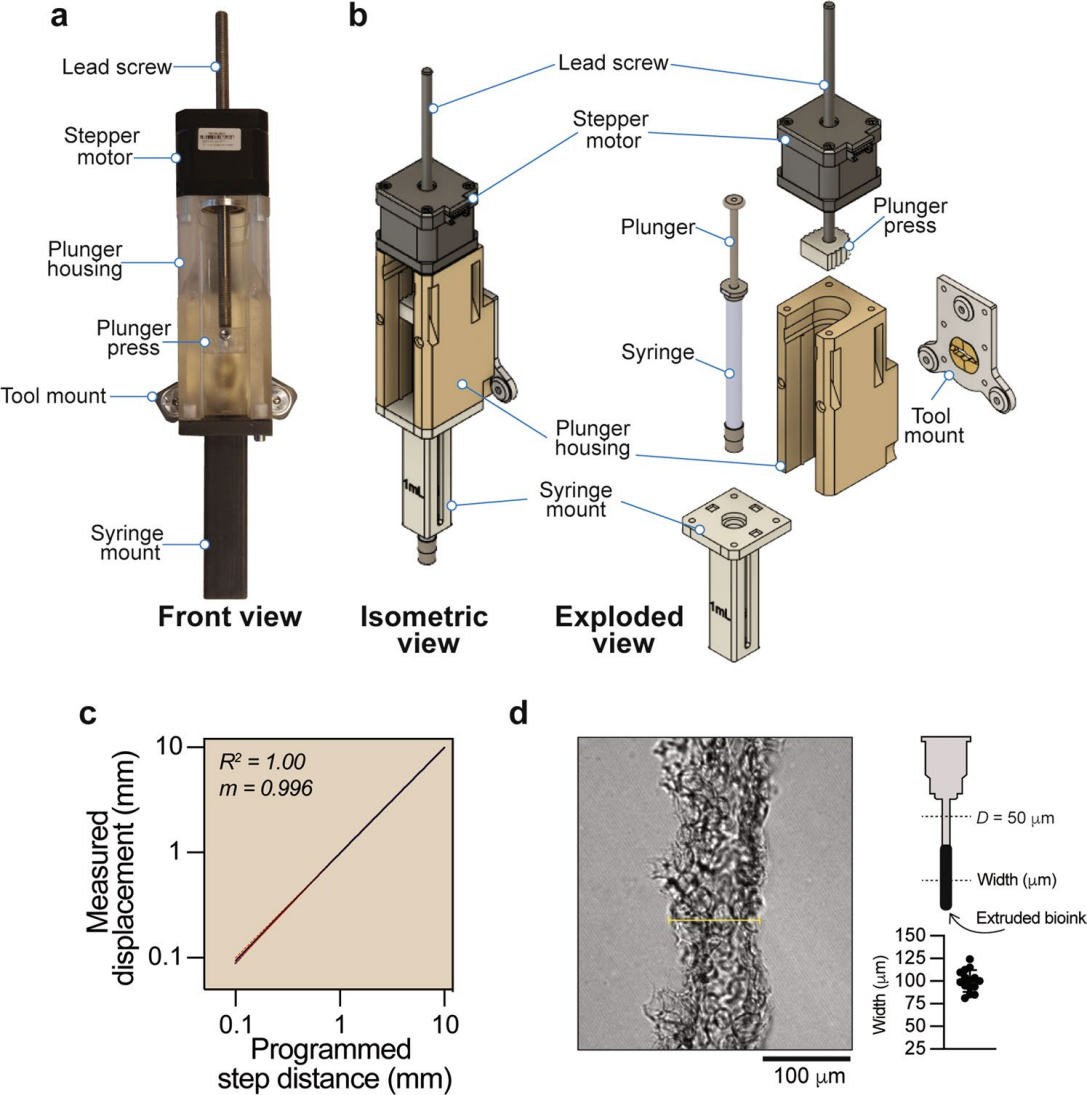
Design of the syringe pump extrusion tool. (**a**) A photograph of the assembled syringe pump extrusion tool. The syringe plunger is pushed down by a lead screw via a plunger press. The lead screw is linearly translated along the z-axis through a stepper motor, which is attached atop the 3D printed plunger housing. 3D printed exchangeable syringe mounts are attached to the base of the plunger housing, and the entire syringe pump extrusion tool is attached to the tool exchanger system via the E3D tool mount fixture. (**b**) Isometric and exploded views of CAD illustrations of the syringe pump extrusion tool. (**c**) Linear regression analysis comparing the vertical displacement (mm) of the syringe plunger as measured by a dial indicator with the intended step sizes 10 mm, 1 mm or 0.1 mm set on the stepper motor. Ten measurements were recorded for each step size (red dotted lines indicate S.D.). (**d**) A differential interference contrast image detailing a section of a collagen string bioprinted using the FRESH protocol through a needle with a cross-sectional diameter of 50 μm. Scatterplot of width measurements recorded from multiple locations along the length of the printed string (100 ± 12 μm).

The custom-built syringe pump extrusion tools were built around a stepper motor that linearly translates an integrated lead screw (Fig. 2a). The stepper motor was mounted on top of a 3D-printed plunger housing with an attached E3D tool mount at the back of the housing (Fig. 2a,b). The end of the lead screw was fitted with a plunger press (most clearly seen in Fig. 2b, exploded view), which applies force to the syringe plunger to extrude bioinks (hydrogels) from the syringe. The plunger housing was in turn attached to the top of a 3D-printed syringe mount (Fig. 2b). The syringe mounts can easily be designed to hold either 0.25, 1, or 5 ml Hamilton gastight syringes, or 5 ml BD syringes, and are compatible with the extrusion of bioinks through different needles or nozzle attachments. Notably, the minimum volume of dispensed bioink is dependent on both the inner diameter of the selected syringe and the micro-stepping setting of the stepper motor. To assess the accuracy with which the stepper motor vertically displaced the syringe pump tool, a dial test indicator was mounted in place of a syringe and the actual distance displaced was recorded when the stepper motor step size was set at 10 mm, 1 mm, and 0.1 mm (Fig. 2c). By accounting for the recorded deviation in actual displacement distance (0.094 ± 0.005 mm) when the stepper motor was set to depress 0.1 mm, we calculated that a 0.25 ml Hamilton syringe will extrude 0.39 ± 0.02 μl (mean ± S.D.) of bioink. The syringe mounts were equipped with three adjustable thumbscrews, which allowed the syringe to be centrally positioned in the mount to ensure perpendicular alignment to the build platform. Importantly, the custom built syringe pump presented here shares similarities with and is in part adapted from previously proposed solutions^22^, where the versatility afforded by accommodating different syringe types has also been demonstrated^23^. We additionally tested the capacity of the bioprinter to accurately reproduce a specific construct feature. Strings of collagen bioink were extruded using FRESH bioprinting through a 50 μm needle and imaged. The cross-sectional width was measured from multiple locations along the length of a printed string using ImageJ, and the average line width was determined to be 100 ± 12 μm (Fig. 2d).

### FRESH bioprinting of collagen constructs

**Figure 3:**
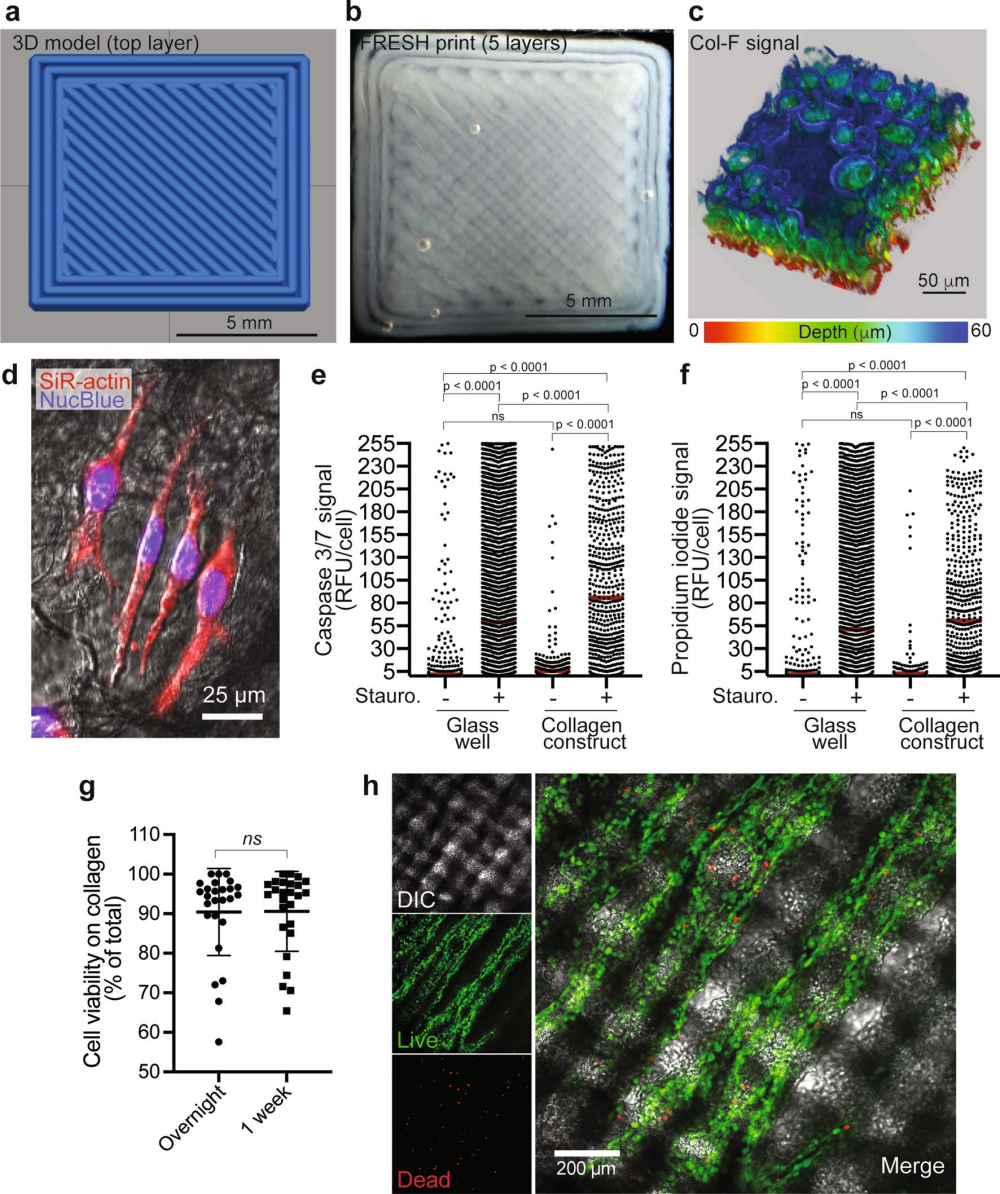
FRESH bioprinting using the open source bioprinter. (**a**) Design of the construct to be printed using FRESH methodology, and (**b**) actual FRESH bioprinted construct. (**c**) 3D rendering of a Col-F stained bioprinted collagen construct based on imaging by confocal microscopy. (**d**) Example of MDA-MB-231 breast cancer cells seeded onto a bioprinted collagen construct post printing. The cells were stained with SiR-actin and NucBlue to visualize the actin cytoskeleton and cell nuclei respectively. The cells were shown to attach and adopt normal cell morphologies a few hours after cell seeding. The relative level of active apoptosis (**e**) as measured by Caspase 3/7 activity, and relative occurrence of dead cells (**f**) as measured by propidium iodide signal in cells grown in glass-bottomed wells or on FRESH bioprinted collagen constructs, in the presence or absence of the apoptosis-inducing toxin staurosporine (10 μM for 5 h) are shown. (**g**) Analysis of cell viability as assessed by fluorescein diacetate (live) and propidium iodide (dead) staining of MDA-MB-231 breast cancer cells after overnight culture (~ 20 h) on bioprinted collage constructs, and 1 week later. (**h**) Example of fluorescein diacetate (live cells) and propidium iodide (dead cells) staining of MDA-MB-231 breast cancer cells distributed on the bioprinted collagen constructs following 1 week of culture. Differential interference contrast imaging (DIC) reveals the crisscross patterning of the construct.

As the FRESH bioprinting method has been widely adopted by the bioprinting research community, we assessed its implementation on the present open source bioprinter platform. A cuboid construct was designed using Autodesk Fusion 360. It consisted of five square layers with a tool path that printed the infill as a continuous line, which for each layer followed a series of diagonal, parallel paths (Fig. 3a). The direction of the diagonal print path rotated by 90° between layers, which produced the crisscrossed lattice appearance seen in the final FRESH printed part, which had similar dimensions to those of the intended design (Fig. 3b). The construct was printed using a collagen-based bioink (Collagen Ink 240) in a FRESH LifeSupport gelatin support bath. To facilitate ease of handling, the construct was printed within a custom-made 3D printed polyetherketoneketone (PEKK) basket, which was manually removed from the support bath to permit transfer of the construct for melting of the gelatin microparticles, and subsequent washing of the released construct (Fig. 3b). It has previously been demonstrated that the gelatin microparticles in the FRESH support bath imprint topological indentations in the collagen surfaces of the printed constructs^16^. Here, confocal microscopy z-stack imaging of Col-F stained FRESH bioprinted constructs confirmed the presence of similar microparticle dimples on the surface of the collagen constructs produced using the open source bioprinter (Fig. 3c). These depressions are likely advantageous for cell seeding as they present craters on the surface of the constructs, which trap cells seeded in suspensions and promote cell attachment followed by infiltration of the constructs.

### Viability of cells grown on bioprinted collagen constructs

Individual FRESH bioprinted collagen constructs were moved into wells of a chambered coverslip with a glass bottom compatible with high resolution microscopy. MDA-MB-231 breast cancer cells were seeded onto the collagen constructs, or for comparison were seeded into wells without constructs, where after the cells were cultured for 20 h. Staining of cells to visualize the cytoskeleton as well as cell nuclei identified cells that had attached to the collagen construct and adopted normal cell morphology (Fig. 3d). To assess the viability of cells seeded in the bioprinted collagen constructs, as compared to cells grown in conventional glass bottom culture chambers, cells were incubated with the Cellevent caspase 3/7 detection reagent that reports on apoptosis, and propidium iodide, which detects the presence of dead cells, and individual cells were identified by the nuclear stain NucBlue. To confirm that this imaging strategy for assessing viability was reliable in the context of the collagen constructs, we additionally treated cells with the potent kinase inhibitor staurosporine^24^, known to induce cell death via apoptosis^25,26^. Cells treated with staurosporine served as positive controls for the Cellevent caspase 3/7 and propidium iodide fluorescence. Importantly, there was no difference between the degree of apoptosis (Fig. 3e) or presence of dead cells (Fig. 3f) in cells grown in the glass-bottomed wells compared to those seeded into the bioprinted collagen constructs. We further assessed cell viability 1 week after cells were seeded onto the collagen constructs, using a live/dead staining protocol employing propidium iodide and fluorescein diacetate. We found viability to be similar between overnight (~ 20 h) and 1 week cultures, which was on average 90% (Fig. 3g). Interestingly, the crisscross patterning of the printed collagen constructs was occasionally observed to influence the distribution of the seeded cells, such that they appeared to align with the topology of the printed collagen strings in the construct (Fig. 3h). Together this data demonstrates that the FRESH collagen constructs produced using this bioprinter are compatible with cell seeding and long-term cell survival.

### Viability of cells extruded in laminin bioink

**Figure 4:**
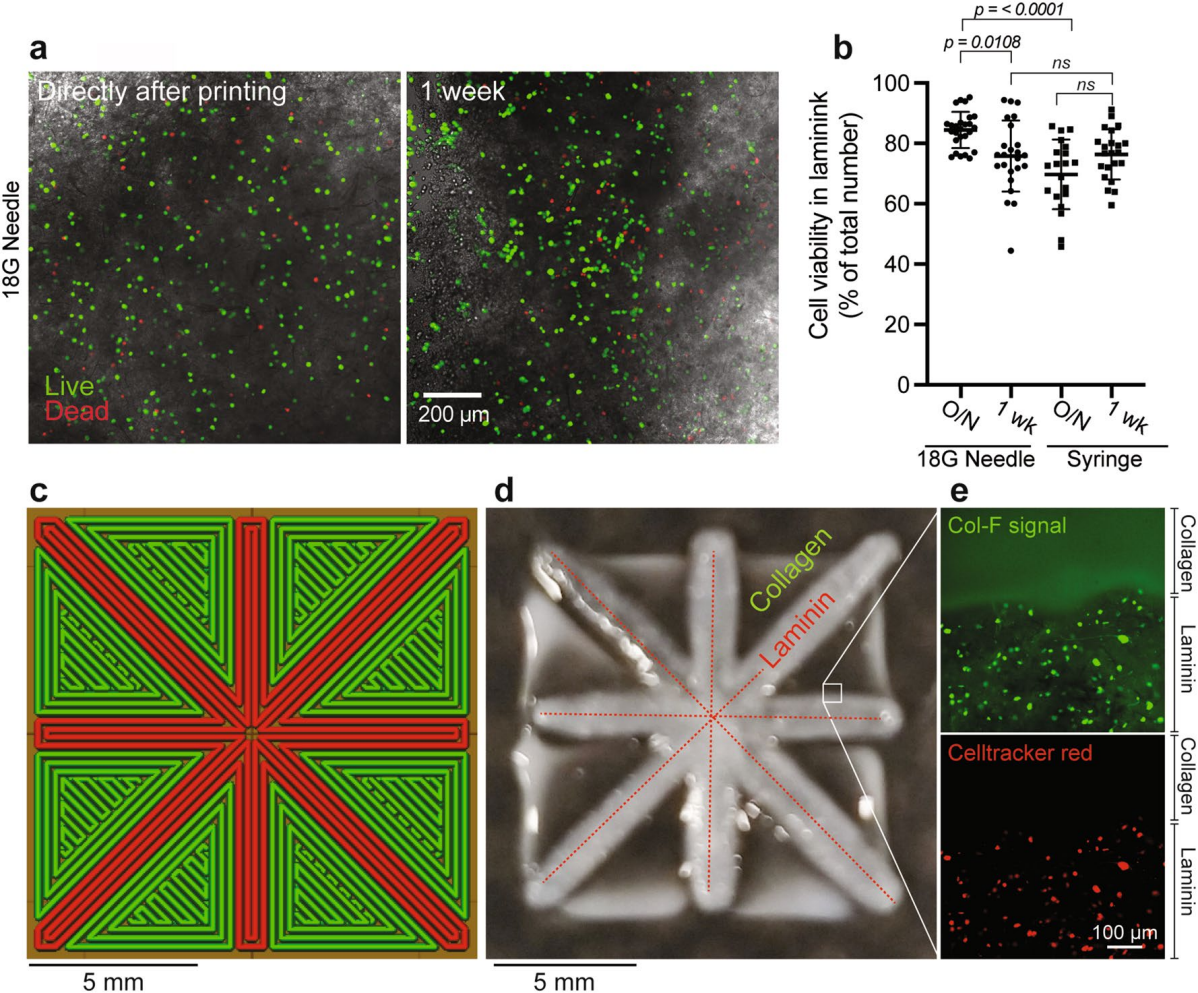
Multimaterial bioprinting using the open source bioprinter. (**a**) Example of fluorescein diacetate (live cells; green) and propidium iodide (dead cells; red) staining of MDA-MB-231 breast cancer cells extruded in laminin bioink directly after extrusion through an 18G needle and following 1 week of culture. (**b**) Comparative analysis of cell viability following overnight culture (O/N) and one week after extrusion of cells in laminin bioink through an 18 G needle or manually deposited directly from a syringe. (**c**) Design of the multimaterial construct to be printed using both collagen (green) and cell-laden laminin (red) bioinks, as viewed from above illustrating the print path. (**d**) Image of the bioprinted multimaterial construct. (**e**) Col-F staining of the collagen bioink (green) and Celltracker red staining of MDA-MB-231 cells distributed in the laminin bioink.

Extrusion of cell-laden bioinks circumvents certain problems associated with the seeding of constructs with cells post bioprinting. Firstly, the distribution of cells in the construct is likely homogenous, and at least for thicker constructs, the ability of cells to penetrate into the depths of such constructs will not be a limiting factor, which may be an issue when studying cell-types that have a limited capacity to migrate. In addition, it is difficult to precisely control the spatial distribution of different specific cell types in a complex construct by cell seeding after bioprinting. Instead, constructs can be composed of independently extruded hydrogels each containing specific cell types of interest. However, extrusion of cell-containing hydrogels exerts shear stress on the cells and can reduce cell viability^12^. Here, we determined to assess the viability of MDA-MB-231 cells after they had been extruded in laminin bioink through an 18 G needle as compared to the viability of cells that had been manually deposited in the bioink through a syringe. Cell viability was assessed by the live/dead protocol as described above, and viability was scored the day after (~ 20 h) printing or extrusion through the syringe and 1 week later (Fig. 4a,b). The highest cell viability was observed the day after printing through the 18G needle, which declined by approximately 10% after one week of culture (Fig. 4b). Viability was initially lower for cells that were manually deposited directly from the syringe, but similar levels of viability were observed after 1 week for both modes of deposition (Fig. 4b), suggesting that the printing process itself does not negatively impact on cell viability in the laminin constructs, and yields cell-laden constructs useful for long-term studies. In contrast to the relatively spread morphology observed for cells on the collagen constructs, cells appeared rounded in the laminin prints. As MDA-MB-231 breast cancer cells are neoplastic they engage in anchorage-independent growth, and due in part to differences in their pattern of integrin expression these cells bind more readily to collagen than laminin^27^. Benton et al. similarly observed that MDA-MB-231 cells transition from a spindle-like morphology on collagen, to a more rounded morphology in laminin, which lead to the formation of cell clusters^28^. Therefore, while the MDA-MB-231 cells may not readily attach and spread in the laminin constructs, they were well distributed and viable.

### Multimaterial bioprinting

The capacity of a bioprinter to produce constructs composed of two or more materials presents important advantages for construct design. For example, combining a network of sacrificial materials (e.g. Pluronic F127) into a hydrogel construct, which is removed after printing, can provide perfusion channels, which can increase cell viability and survival in larger constructs^29^. Furthermore, combining bioinks in controlled ratios to reproducibly create experimental tissue culture environments, enables studies of cell responses in which the role of the surrounding matrix composition or specific treatments can be evaluated^30–33^. To demonstrate the multimaterial printing capacity of the open source bioprinter, we designed a square construct intersected by eight spokes originating from the constructs center, to be printed with cell-laden laminin bioink (Fig. 4c, red lines). The spaces between the laminin spokes and the outer boundary of the square were filled with collagen bioink (Fig. 4c green lines). The final print had similar dimensions to the intended design (Fig. 4d). Col-F staining of the collagen ink revealed a defined border between the collagen and laminin regions of the print (Fig. 4e). Unexpectedly, Col-F also stained the MDA-MB-231 cells in the laminin regions of the construct, and like the Celltracker red staining revealed an even distribution of the cells in the laminin bioink (Fig. 4e).

## Conclusion and outlook

The open source bioprinter presented here, based on the E3D motion system and tool changer, enables a variety of bioprinting tasks including FRESH bioprinting, multimaterial prints, and the extrusion of cell-laden bioinks. In the presented configuration only two of the four tool positions were used. The remaining tool positions present opportunities for further automation of protocols to reduce user intervention, and to increase reproducibility and throughput. For instance, we are currently developing a grip tool and protocol to manipulate the 3D printed basket in which the FRESH collagen constructs are bioprinted, to allow for automated construct transport from the gelatin support bath to a heated washing station, which melts the gelatin support material. The grip tool can subsequently move the construct through additional washing solutions, effectively automating key steps of the post-processing procedure. The benefits of automating biofabrication protocols have been outlined for the formation of tissue spheroids^34,35^, the analysis of multi-cell type behaviors^36^, and is important for future efforts to facilitate and scale up bioprinting of constructs for use in research and drug screening. The open source bioprinter is easily adapted for different bioprinting applications, and the possibility of incorporating additional tools should accelerate the development of novel bioprinting approaches and applications.

## Materials and methods

### The open source bioprinter and syringe pump extrusion tool

The bioprinter (Fig. 1) was built around the E3D tool changer and motion system (E3D-online, London, United Kingdom)^20,21^, and enclosed in a custom-built polycarbonate cabinet with an integrated a HEPA filter with an air intake fan. The syringe pump extrusion tool (Fig. 2) was built using a stepper motor, a lead screw, and 3D-printed plunger housing and syringe holder. All computer-aided design (CAD) was done using Fusion 360 (Autodesk Inc, San Rafael, USA). All 3D-printed parts for the extrusion tools were printed on a Form 3 SLA 3D printer (Formlabs) in standard clear resin or tough 2000 resin (Formlabs) with a Z-resolution of ~ 100 µm. All prints with resin were washed and cured according to the manufacturer’s instructions. The baskets used for handling FRESH-printed constructs were 3D-printed in PEKK-A (Kimya, Nantes, France) on a miniFactory Ultra FFF 3D-printer (miniFactory Oy LTD, Seinäjoki, Finland). Brackets for the enclosure, camera housing, touch screen mount and HEPA-filter holder were printed in PLA (Add North 3D AB, Ölsremma, Sweden) on a Prusa I3 MK3S (Prusa, Prague, Czech Republic). STL-files for all 3D printed components as well as a full list of all components required to build the bioprinter and syringe pump extrusion tool are included in the Supplementary Materials section.

### Bioprinter operation

Before powering up the printer, the stepper motors driving the syringe pump extrusion tools were connected to the control board and the tools placed in the tool dock. The E3D motion system is controlled by a Duet Wifi controller board together with the expansion board Duex 5. This enables control of up to 10 stepper motors. 4 stepper motors in the E3D motion system are used to control the x-, y- and z-axes as well as the tool changer mechanism. In addition, syringe pump extrusion tools use one stepper motor output each. Configuration of any additional tools are made via modification of the *config.g-file* in the DWC interface. For multimaterial prints, requiring the use of two or more tools, calibrations for tool offsets were required as described below.

### Tool offset calibration

Tool offset identification was performed using the “Tool Alignment with Machine Vision” (TAMV) program originally developed by Danal Estes^37^, using a Logitech web camera mounted on the print platform and a Raspberry Pi 4B running OpenCV. A prerequisite for running the calibration commands is that all axes first are homed. The procedure for x- and y-axis tool offset identification is performed per the instructions on the creator’s website^37^. Z-axis offset calibration is facilitated by a microswitch mounted on the build plate, this enables probing of individual tools to identify differences in syringe and needle length. For the calibration to work correctly the leadscrew in the stepper motor must be engaged with the plunger via the plunger press (Fig. 2). This is done by running the “Tool 0—Select” macro from the “macros” section of DWC, followed by stepwise movement of the leadscrew until it applies pressure to the plunger, which is repeated for each syringe pump tool. The procedure for z-axis offset calibration is performed automatically via a custom-made G-code script, using the spatial positioning of the first tool as the reference for offset calibration for the other tools. The first tool is picked up and positioned with the needle tip above the microswitch, followed by movement of the build plate until the microswitch is engaged. After the reference position of the first tool has been logged, other tools are then picked up and used to trigger the microswitch. At the end of the procedure, a z-offset value is presented in the DWC web interface and can be inserted manually into *config.g* which stores the offset values for each tool. This procedure is repeated between each new print since needle length and position of needle tips in the tool will differ between prints. Once calibration is complete bioprinting can be initiated by using the macro to pick up the loaded tool, manually positioning it at the starting point, zero the z-axis and start the G-code file for the selected construct of interest.

### Toolpath generation

The toolpath generation software (Simplify3D, Cincinnati, USA) was used to generate G-code files for bioprinting of the different constructs. Bioprinting was typically performed at a speed of 10 mm/s, as printing at this speed produced reproducible constructs of both the collagen and laminin bioinks. The printing parameters are available in a *.factory* file format, which is native to Simplify3D. Further information about software configuration and slicing settings can be found in the Supplementary Materials section.

### Assessment of stepper motor step accuracy, minimum print volume, and practical print resolution

To determine the accuracy with which the stepper motor vertically displaced a syringe plunger, a dial indicator (Mitutoyo 543-125B, Kanagawa, Japan) was mounted beneath the lead screw and adjusted such that its contact point was in contact with the plunger press. The stepper motor was programmed to take 10 mm, 1 mm, or 0.1 mm steps sizes and for each step the measured displacement distance was recorded from the dial indicator. These measurements were repeated 10 times for each step size, and a linear plot was prepared (Fig. 2c). We calculated the minimum volume of bioink that the bioprinter could extrude through a Hamilton 0.25 ml syringe using the measured displacement values recorded when the step size was set at 0.1 mm, based on the formula for the volume of a cylinder (*πr*^2^*h*), where *r* is the inner radius of the Hamilton 0.25 ml syringe (1.15 mm). This yielded a value of 0.39 ± 0.02 μl (mean ± S.D.). To assess the practical lower limits of the resolution of the bioprinter we used a nozzle with a 50 μm cross-sectional diameter to extrude strings of collagen using FRESH bioprinting. The strings were imaged by differential interference contrast using a Zeiss LSM710 confocal microscope (Zeiss), and the average cross-sectional width of the finest collagen strings that we could produce and recover was calculated from multiple measurements along the length of the printed string (Fig. 2d).

### Cell culture

The MDA-MB-231 breast cancer cell line was obtained from the American Type Culture Collection (ATCC) and cultured in Dulbecco’s Modified Eagle Medium (DMEM) Glutamax (Gibco, Thermo Fisher Scientific, Uppsala, Sweden), supplemented with 10% Fetal Bovine Serum (FBS; Thermo Fisher Scientific), referred to hereafter as culture medium, in a humidified incubator (5% CO_2_, 37 °C) according to the instructions provided by the supplier ATCC.

### Bioink loading

The Collagen Lifeink 240 (Advanced Biomatrix) bioink and LaminInk + (Cellink) laminin bioink were first transferred to a 5 ml plastic syringe with male-male LuerLock. To reduce the presence of bubbles in the bioink, the loaded syringe was centrifuged for 4 min at 1200×*g*, with the aid of a custom-made centrifuge insert. The hydrogel was next transferred to a 1 ml Hamilton gastight syringe via a female-female LuerLock adapter. The syringe was then inserted into the 3D-printed syringe holder, which in turn was bolted into the syringe pump body with four M3 × 10 screws.

### FRESH collagen bioprinting, cell seeding, and construct imaging

FRESH bioprinting was performed according to the protocol by Lee et al.^16^, using LifeInk 240 (Advanced Biomatrix). The FRESH LifeSupport powder (Cellink), from which the gelatin microparticle support bath was prepared, was used as per the manufacturer’s instructions. Following print completion, the tool was left at the tool dock and the build plate lowered. All constructs generated using FRESH bioprinting was made in a custom-designed and easily moveable basket. After a completed print, the basket holding the collagen construct was placed at 37 °C for 1 h to melt the gelatin support material and to thereby release the printed construct. Constructs were washed three times in 1× phosphate buffered saline (PBS, Gibco) and stored in PBS at 4 °C until further use. For fluorescent imaging, collagen constructs were stained for 1 h at 37 °C with 10 μM Col-F (ImmunoChemistry Technologies, Bloomington, USA) prepared in 1× PBS, and then washed twice (5 min each) with 1× PBS at room temperature. Constructs were imaged by confocal microscopy performed on a Zeiss LSM700 (Zeiss, Jena, Germany) instrument and images were captured using Zen imaging software (Zen 2011 SP7 FP3, version 14.00.22.201, Zeiss). For cell seeding, FRESH constructs were first typically stored overnight at 4 °C in 1× PBS supplemented with the antibiotics penicillin and streptomycin (PenStrep; Thermofisher, Uppsala, Sweden). Individual constructs were next moved to separate wells in a µ-slide 8-well coverglass-bottomed chamber (Ibidi) and a cell suspension of 30 × 10^3^ MDA-MB-231 cells diluted in 250 µl culture medium supplemented with 1× PenStrep per well was deposited directly onto the collagen construct or into an adjacent, construct-free wells, and cultured for 20 h under standard cell culture conditions. Cell staining and analysis of viability and cell morphology by microscopy are described below.

### Analysis of cell viability in seeded FRESH collagen constructs

To assess cell viability in FRESH bioprinted collagen constructs, cell-seeded constructs were incubated with the NucBlue nuclear stain to detect individual cells (Invitrogen, Thermo Fisher), propidium iodide (PI) to identify dead cells (1:1000; Invitrogen, Thermo Fisher), and the Cell event caspase-3/7 stain that reports on the activity of caspase 3 and 7 in apoptotic cells (10 μM; Cell event, Invitrogen, Thermo Fisher). Staining solutions were diluted in OptiMEM (Gibco, Thermo Fisher), supplemented with 1× PenStrep. To control for the effectivity of the viability stains, cells were treated for 5 h with staurosporine (10 μM; Abcam, Cambridge, United Kingdom), a potent protein kinase inhibitor known to induce apoptosis. Fluorescent signals for the respective stains were captured by confocal microscopy with an LSM700 instrument and Zen software (Zeiss). Multiple z-planes were captured for cell-laden constructs, while a single in-focus z-plane was captured for cells attached directly to the glass in construct-free wells. These experiments were conducted in duplicate on three independent occasions. Image analysis was conducted in the Fiji version of ImageJ^38^. For cell-laden collagen constructs, the z-plane containing the highest number of cells was selected for analysis. Individual cells were identified as regions of interest (ROI) by applying a threshold to the NucBlue signal. The ImageJ particle analysis function was applied to this thresholded image to include particles within a size range of 15–600 μm^2^. This range excluded non-specific signals from small debris and very large clusters of cells. However, it included smaller cell clusters that could be delineated as individual cells by applying the automatic watershed function to the thresholded image. This ROI mask of individual cells was then overlaid on the PI and Cell event caspase-3/7 channels and the mean fluorescence was recorded in each ROI for each channel. This data was exported to Excel, and mean intensity per ROI and channel was background adjusted using an average from three ROIs measured in cell-free areas of the collagen construct. Data was statistically analyzed using ordinary one-way ANOVA with Tukey’s multiple comparisons in GraphPad Prism (Prism).

### Analysis of cell attachment and cell morphology

To visualize cells adhering to FRESH bioprinted collagen constructs the cells were labelled overnight with SiR-actin (500 nM; Spirochrome, Switzerland), which permits fluorescence imaging of the actin cytoskeleton. Samples were washed twice in OptiMEM cell culture medium, which was then exchanged for OptiMEM containing the NucBlue nuclear stain. The cells were imaged as described above by confocal microscopy.

### Analysis of cell viability in long term culture of cells seeded on collagen

FRESH bioprinting of constructs for one week culture was performed as described above, however to reduce unnecessary handling, constructs were printed directly on a Labtek 8 well chamber coverslip. Onto each construct 24,000 cells/well were seeded in DMEM (10% FBS, 1% PenStrep). Half the media was exchanged every 1–2 days. Live/dead staining was performed on day 1 after cell seeding, and in separate constructs 1 week after cell seeding. Briefly, cells were stained with fluorescein diacetate (Sigma) 8 µg/ml and PI, diluted 1:1000 in OptiMEM, for 10 min. Then constructs were washed once with OptiMEM. Images were taken using an LSM 700 confocal microscope (Zeiss) and a 10× objective. For each construct two images were taken of a z-plane with high cell density and used for analysis in ImageJ. In ImageJ both channels were thresholded manually and the automatic watershed function applied. Subsequently, the particle analysis tool was used to count the number of live or dead cells. Using Excel, the percentage of viable cells was calculated for each image. To determine statistically significant differences between groups, data were imported to GraphPad Prism and analyzed by Student’s t-test. Experiments were performed in three independent repetitions with two timepoints and four to five constructs per timepoint and repetition.

### Analysis of cell viability following extrusion of cell-laden laminin bioink

Cell-laden laminin bioink was prepared as follows; 100 µl of cell suspension containing 11 × 10^6^ cells/ml was mixed with 1 ml Laminink + Bioink (Cellink) by passing back and forth between two 1 ml syringes. For control, the cell-laden bioink was directly deposited into wells from the plastic syringe without extrusion through a thin needle or nozzle. The remaining ink was loaded into a 250 µl Hamilton glass syringe and bioprinting performed with an 18 G needle. For crosslinking of all constructs, including non-printed controls, the recommended Crosslinking Agent (Cellink) was applied for 1 min. Subsequently, the constructs were washed once in warm (37 °C) DMEM Glutamax, supplemented with 10% FBS and 1× PenStrep, and then either cultured for one week in 100 µl of media, with half the media being exchanged daily, in a cell incubator (5% CO_2_, 37 °C) or directly used for live/dead staining, described above. Experiments were conducted in three independent repetitions with two timepoints and a total number of three to five printed constructs and non-printed control hydrogels for each group and repetition. For each construct two images were taken of a z-plane with high cell density and used for particle analysis in ImageJ, and statistical analysis in GraphPad Prism (Prism), as described above.

### Multimaterial bioprinting with collagen and laminin bioinks

Multimaterial constructs were bioprinted using acidified collagen (Advanced Biomatrix) and Laminink + (Cellink) containing cells as described above. Printing was done directly onto a glass slide, followed by incubation in 50 mM HEPES (pH 7.4) containing 50 mM CaCl_2_, to induce gelation of collagen and laminin, respectively.

## Supplementary Information

Supplementary Information 1.

Supplementary Information 2.

Supplementary Information 3.

Supplementary Information 4.

## Data Availability

The datasets generated and analyzed are available on request. STL files, scripts and a bill of materials are included as Supplementary Information.
