# Supplementary material for: An open source extrusion bioprinter based on the E3D motion system and tool changer to enable FRESH and multimaterial bioprinting
Source: Sci Rep. 2021 Nov 3;11:21547. doi: 10.1038/s41598-021-00931-1 (PMC8566469; doi:10.1038/s41598-021-00931-1)
Supplement: Supplementary file 4 — Supplementary Information 4. [file 41598_2021_931_MOESM4_ESM.docx]

**Supplementary information**

**An open source extrusion bioprinter based on the E3D motion system and tool changer to enable FRESH and multi-material bioprinting**

Adam Engberg^1^, Christina Stelzl^1^, Olle Eriksson^1^, Paul O’Callaghan^1^, and Johan Kreuger^1*^

^1^Department of Medical Cell Biology, Uppsala University, Uppsala, Sweden

*Corresponding author

E-mail: johan.kreuger@mcb.uu.se

**Supplementary data 1. Bill of materials.**

**Supplementary data 2. Printer components, STL and STEP files.**

**Supplementary data 3. Slicing profiles, configuration files, and macros.**
